# Comparative psychometric analyses of the SCL-90-R and its short versions in patients with affective disorders

**DOI:** 10.1186/1471-244X-13-104

**Published:** 2013-03-28

**Authors:** Ulrich Prinz, Detlev O Nutzinger, Holger Schulz, Franz Petermann, Christoph Braukhaus, Sylke Andreas

**Affiliations:** 1 Center of Psychiatric Care Rickling, Rickling, D - 24635, Germany; 2 Psychosomatic Clinic, Bad Bramstedt, Bad Bramstedt, D-24576, Germany; 3 Department of Medical Psychology, University Medical Centre Hamburg-Eppendorf, D-20246 Hamburg, Germany; 4 Center for Clinical Psychology and Rehabilitation, University of Bremen, Bremen, D-28359, Germany; 5 Institute of Psychology, University of Klagenfurt, A-9020 Klagenfurt, Austria

**Keywords:** SCL-90-R, Short versions, Psychometric, Affective disorder, Symptom severity

## Abstract

**Background:**

Despite the widespread application of Symptom Checklist 90-R (SCL-90-R), its psychometric weaknesses have repeatedly been noted. This study aimed to comparatively assess the psychometric properties of the SCL-90-R scales and the scales of its short versions Brief Symptom Inventory (BSI), Symptom Checklist-27 (SCL-27), Brief Symptom Inventory-18 (BSI-18), Symptom Checklist-14 (SCL-14), and Symptom Checklist short version-9 (SCL-K-9) in patients with affective disorders.

**Methods:**

The data of 2,727 patients within the main treatment group of affective disorders were assessed according to the DSM-IV. Patients completed the SCL-90-R and Beck Depression Inventory (BDI).

**Results:**

There were no significant differences regarding the internal consistency of the SCL-90-R scales and the scales of the short versions. The dimensional structure was only supported for the short versions BSI-18, SCL-14 and SCL-K-9. The assessment of convergent validity revealed high correlations. With regard to the discriminant validity, there were medium correlations. With regard to the sensitivity of change, no significant differences between the scales were found.

**Conclusions:**

In summary, the scales of the short versions show mostly satisfactory psychometric properties in comparison to the scales of the SCL-90-R. The results support the application of the short versions as screening instruments, especially the BSI-18, and more economic variants of the SCL-90-R covering a wide range of psychopathological symptoms.

## Background

The Symptom Checklist 90-R (SCL-90-R) [1] is a widely applied self-assessment instrument for a broad range of mental disorders that assesses the subjective symptom burden in patients with mental disorders. Alongside the high acceptance and extensive worldwide application of the instrument as an outcome instrument in the treatment of patients with affective disorders, a number of studies have highlighted the psychometric weaknesses of the SCL-90-R. A central criticism is that many studies were unable to replicate the postulated factor structure and instead found a general factor with large variance [2,3]. This finding is relevant with regard to the practicality of the instrument because results on the factorial validity suggest partial redundancy of the collected data.

The development of numerous short versions of the SCL-90-R indicates the need for a more economic instrument. The short versions of the SCL-90-R [4] that have been developed include either a reduced number of items referencing the postulated factor structure, such as the Brief Symptom Inventory (BSI-53) [5] and Brief Symptom Inventory-18 (BSI-18) [6], or a reduced item number with a reduced factor structure, such as the SCL-27 [7] and SCL-14 [8]. Other SCL-90-R short versions with a considerably reduced item number measure only a general severity factor, such as the SCL-K-9 [9,10]. Table 1 gives an overview of each short version with their respective scales and item positions compared to the SCL-90-R.

**Table 1:** Dimensional structure of the SCL-90-R and its short versions BSI, SCL-27, BSI-18, SCL-14 and SCL-K-9

**Questionnaire**	**Dimension**	**Number of items**	**Item position in the SCL-90-R**
SCL-90-R	Somatization	12	1, 4, 12, 27, 40, 42, 48, 49, 52, 53, 56, 58
	Obsessive compulsive	10	3, 9, 10, 28, 38, 45, 46, 51, 55, 65
	Interpersonal sensitivity	9	6, 21, 34, 36, 37, 41, 61, 69, 73
	Depression	13	5, 14, 15, 20, 22, 26, 29, 30, 31, 32, 54, 71, 79
	Anxiety	10	2, 17, 23, 33, 39, 57, 72, 78, 80, 86
	Hostility	6	11, 24, 63, 67, 74, 81
	Phobic anxiety	7	13, 25, 47, 50, 70, 75, 82
	Paranoid ideation	6	8, 18, 43, 68, 76, 83
	Psychoticism	10	7, 16, 35, 62, 77, 84, 85, 87, 88, 90
	Additional items	7	19, 44, 59, 60, 64, 66, 89
BSI	Somatization	7	4, 12, 40, 48, 49, 52, 56
	Obsessive compulsive	6	9, 28, 45, 46, 51, 55
	Interpersonal sensitivity	4	34, 37, 41, 69
	Depression	6	15, 29, 30, 32, 54, 79
	Anxiety	6	2, 23, 33, 57, 72, 78
	Hostility	5	11, 24, 63, 67, 74
	Phobic anxiety	5	13, 47, 50, 70, 75
	Paranoid ideation	5	8, 18, 43, 76, 83
	Psychoticism	5	7, 77, 85, 88, 90
	Additional items	4	19, 44, 59, 89
SCL-27	Depressive symptoms	4	15, 30, 54, 59
	Dysthymic symptoms	4	9, 14, 51, 55
	Vegetative symptoms	6	4, 39, 40, 48, 49, 53
	Agoraphobic symptoms	5	13, 25, 33, 50, 82
	Symptoms of social phobia	4	37, 41, 61, 69
	Symptoms of mistrust	4	18, 68, 76, 83
BSI-18	Somatization	6	12, 40, 48, 52, 56, 58
	Depression	6	15, 29, 30, 32, 54, 79
	Anxiety	6	2, 33, 57, 72, 78, 86
SCL-14	Depression	6	26, 28, 30, 54, 77, 79
	Phobic anxiety	4	13, 25, 47, 82
	Somatization	4	42, 52, 56, 58
SCL-K-9	Global Severity Index	9	24, 28, 31, 34, 43, 57, 58, 75, 77

Notes: SCL-90-R = Symptom Checklist 90-R [4]; BSI = Brief Symptom Inventory [6]; SCL-27 = Symptom Checklist-27 [7]; BSI-18 = Brief Symptom Inventory 18 [6]; SCL-14 = Symptom Checklist 14 [8]; SCL-K-9 = Symptom Checklist-short version-9 [10].

There are some comparative studies available assessing the validity of individual short versions in different settings. One study by Müller et al. (2010) examined the psychometric properties of eleven short versions of the SCL-90-R in a sample of 100 German mothers of young children treated at a child psychiatric family day clinic. Müller et al. [11] concluded that compared to the other short versions, the SCL-10S is a good screening instrument, but further research on larger samples is needed. Another study conducted by the authors of this paper [12] more generally examined the psychometric properties of short versions of the SCL-90-R in a large sample (N = 8,581) of inpatients with various mental disorders. Compared to the subscales of the SCL-90-R, the subscales of the short versions of the SCL-90-R showed satisfactory psychometric properties in various mental disorders. The results supported the implementation of the short versions BSI, SCL-27, SCL-14, and - within limits - SCL-K-9 as suitable screening instruments to assess a wide range of psychopathologic symptoms.

Empirical data from health surveys have shown that the prevalence rate of affective disorders ranges from 6.6% [13] to 11.9% [14]. Not only are affective disorders a highly prevalent disorder in the general population, people with affective disorders comprise one of the largest patient groups in inpatient and outpatient psychotherapeutic settings [15]. No empirical data on the appropriateness of the SCL-90-R and its short versions within this large diagnosis group exist.

Therefore, the aim of this study was to assess the psychometric properties (reliability, convergent and discriminant validity, and sensitivity to change) of the SCL-90-R scales (including the global severity index, GSI) and the scales of the short forms (BSI, SCL-27, BSI-18, SCL-14, and SCL-K-9) in patients with *affective disorders*. This study should also add substantial information on the *concurrent validity* of the SCL-90-R and its short versions in patients within the most prevalent subgroup of affective disorders.

## Methods

### Design and setting

This study was conducted at one of the largest clinics for inpatients with mental disorders in Germany between 2000 and 2003. The clinic concept comprises mainly cognitive-behavioural therapy. Patients (N = 2,727) were consecutively included in the study after providing written informed consent. The study was conducted in line with the Code of Ethics of the World Medical Association (Declaration of Helsinki, 1964) and approved by the institutional review board of the Schoen Hospital Company.

The patients routinely completed the SCL-90-R and the Beck Depression Inventory (BDI) [16] at the beginning and end of treatment. The average treatment duration was 48 days (SD = 18 days). Post-hoc, the items of the BSI, SCL-27, BSI-18, SCL-14, and SCL-K-9 were extracted from the long version of the SCL-90-R.

### Sample

The mean age of the sample was 51 years (SD = 12 years, range 23–91 years): 67% of the patients were female, 49% were married, 30% were single, and 17% were divorced. The largest group of patients had German-equivalent secondary school-level education, 31% had CSE level, and 25% had A-level education.

The therapists diagnosed the patients according to DSM-IV [17] criteria at the end of treatment. Most of the patients suffered from recurrent major depressive disorder (n = 1,606, 59%), followed by major depressive disorder, single episode (n = 922, 34%), dysthymic disorder (n = 130, 5%), and depressive disorder not otherwise specified (n = 69, 3%).

### Instruments

The instruments applied in this study were the German version of the SCL-90-R [18] and the German version of the Beck Depression Inventory [19], a widely applied and well-known disorder-specific self-report instrument that assesses the severity of depression symptoms. Retaining the five-level Likert scale from >> 0 = none at all to 4 = very severe, the following short versions were extracted from the SCL-90-R scales post-hoc (see Table 1): BSI [18], BSI-18 [6], SCL-27 [7], SCL-14 [8], and SCL-K-9 [10].

### Statistical analyses

With regard to reliability, the internal consistencies of the subscales of the SCL-90-R and the subscales of the short versions (BSI, SCL-27, BSI-18, SCL-14, and SCL-K-9) were calculated using the Cronbach´s alpha reliability coefficient. Cronbach´s alpha values of α ≥ 0.70 are interpreted as satisfactory [20]. All coefficients of the SCL-90-R scales and scales of the corresponding short forms (BSI, SCL-27, BSI-18, SCL-14, and SCL-K-9) were assessed for significant differences based on Fisher´s z-tests.

To assess the factorial validity of the SCL-90-R scales and scales of the short forms (BSI, SCL-27, BSI-18, SCL-14, SCL-K-9), confirmatory factor analyses (CFAs) were calculated using the AMOS 4.0 software package (Analysis of Moment Structures) [21]. Confirmatory factor analysis was conducted to assess whether the scales postulated by the test authors could be replicated. Moreover, fit indices (standardised root mean square residual (SRMR), root mean square error of approximation (RMSEA), and comparative fit index (CFI)) were calculated according to Hu and Bentler (1999). The recommended cut-off values were the following: RMSEA ≤ 0.06, SRMR ≤ 0.08 (or 0.10), and CFI ≥ 0.95 [22]. Missing data (5% total missing data at time point t1) were replaced using the expectation-maximisation algorithm (EM algorithm) [23].

The mean value differences and correlations were calculated to assess the equivalence of the SCL-90-R and its short versions. It was expected that the scales and the total score of the short versions would on the one hand significantly differ from and on the other hand correlate highly with the scales and the total score of the SCL-90-R long version. To assess the size of the corresponding mean value difference, effect sizes were calculated as follows: *d*_*effect size*_ = *M*_*pre*_–*M*_*post*_/*SD*_*pooled*_[24].

To assess the convergent validity correlations, Pearson product–moment correlations were calculated between the depression scale of the SCL-90-R, the depression subscales of the short versions (BSI, SCL-27, BSI-18, SCL-14, and SCL-K-9) and the BDI total score. Therefore, significant correlations with large effect sizes were expected. The size of the correlations was based on the following interpretation limits according to Cohen [24]: 0.10 > r < 0.30, small effect size; 0.30 > r < 0.50, medium effect size and r > 0.50, large effect size.

To assess the discriminant validity correlations between all other subscales of the SCL-90-R and its subscales with the BDI, the total score was calculated. It was expected that the correlation between the SCL-90-R and the short version scales with the total BDI score would show only small effect sizes (r < 0.30).

To assess the sensitivity to change, the mean value differences in SCL-90-R scales and the short version scales (BSI, SCL-27, BSI-18, SCL-14, and SCL-K-9) between patient admission to and discharge from the clinic were calculated. Cohen’s d [24] was chosen as a measure of the effect size and calculated according to the following formula: *d*_*effect size*_ = *M*_*pre*_–*M*_*post*_/*SD*_*pre*_. The effect sizes were interpreted as follows: 0.20 > d < 0.50, small effect size; 0.50 > d < 0.80, medium effect size and d > 0.80, large effect size.

## Results

### Reliability of the short forms in comparison to the SCL-90-R

Internal consistencies were calculated to assess reliability. Overall, the internal consistencies of the SCL-90-R scales ranged from α = 0.74 (“aggression”) to α = 0.97 (GSI) and were thus regarded as satisfactory. The values of the BSI scales ranged from α = 0.67 (“aggression”) to α = 0.96 (GSI) and were thus, on average, slightly lower than those of the SCL-90-R but could also be regarded satisfactory. A similar picture emerged for the SCL-27 scales (α = 0.73 “mistrust” to α = 0.93 “GSI”). The internal consistencies of the BSI-18 and SCL-14 scales showed consistently satisfactory results (BSI-18: α = 0.79 “somatisation” to α = 0.90 “GSI”; SCL-14: α = 0.81 “somatisation” to α = 0.88 “GSI“), which was similar to the GSI of the SCL-K-9 (α = 0.87). No significant differences were found between the internal consistencies of the compared scales in a psychometric examination of the calculated coefficients using Fisher´s z-test based on the large sample size used (see Table 2).

**Table 2:** Comparison of internal consistencies (Cronbach´s alpha) of the SCL-90-R subscales and the short versions BSI, SCL-27, BSI-18, SCL-14 and SCL-K-9 in patients with affective disorder

**SCL-90-R**			**BSI**			**SCL-27**			**BSI-18**			**SCL-14**			**SCL-K-9**		
**Scale**	**α**	**Number of items**	**Scale**	**α**	**Number of items**	**Scale**	**α**	**Number of items**	**Scale**	**α**	**Number of items**	**Scale**	**α**	**Number of items**	**Scale**	**α**	**Number of items**
SOMA	0.86	12	SOMA	0.80	7	VEG	0.77	6	SOMA	0.79	6	VEG	0.81	4			
OCD	0.87	10	OCD	0.84	6	DYS	0.85	4									
INT	0.87	9	INT	0.79	4	SOP	0.84	4									
DEPR	0.89	13	DEPR	0.85	6	DEP	0.79	4	DEPR	0.85	6	DEP	0.87	6			
ANX	0.87	10	ANX	0.82	6				ANX	0.82	6						
HOST	0.74	6	HOST	0.67	5												
PHOB	0.84	7	PHOB	0.79	5	AGO	0.81	5				AGO	0.81	4			
PARA	0.76	6	PARA	0.74	5	MIS	0.73	4									
PSYC	0.78	10	PSYC	0.70	5												
GSI	0.97	83	GSI	0.96	49	GSI	0.93	27	GSI	0.90	18	GSI	0.88	14	GSI	0.84	9

Notes: Abbreviations of the SCL-90-R, BSI and BSI-18: SOMA = Somatization; OCD = obsessive-compulsive; INT = interpersonal sensitivity; DEPR = Depression; ANX = Anxiety; HOST = Hostility; PHOB = Phobic Anxiety; PARA = Paranoid Ideation; PSYC = Psychoticism; GSI = Global Severity Index; Abbreviations of the SCL-27: DEP = Depressive symptoms; DYS = Dysthymic symptoms; VEG = Vegetative symptoms; AGO = Agoraphobic symptoms; SOP = Symptoms of social phobia; MIS = Symptoms of mistrust; GSI = Global Severity Index; Abbreviations of the SCL-14: DEP = Depression; PHO = Phobic Anxiety; SOM = Somatization; GSI = Global Severity Index; Abbreviations of the SCL-K-9: GSI = Global Severity Index. 2621 ≤ N ≤ 2632.

### Factorial validity of the short forms compared to the SCL-90-R

Table 3 displays the results of the confirmatory factor analysis. Examining the fit indices, the RMSEA (0.06) and the SRMR (0.06) showed a satisfactory model fit for the SCL-90-R scale structure in patients with affective disorders [22]; however, the CFI (0.75) was markedly outside the cut-off limits [22].

**Table 3:** Values of the CFA for the SCL-90-R and the short versions (BSI, SCL-27, BSI-18, SCL-14 and SCL-K-9) in patients with affective disorders

	**Chi-square**			**RMSEA Cut-off < .06**^**a**^	**CFI Cut-off ≈ .95**	**SRMR Cut-off ≤ .11**
	**χ**^**2**^	**df**	**p**			
SCL-90-R	31074.113	3284	.000	.056	.749	.0608
BSI	11443.895	1091	.000	.059	.828	.0536
SCL-27	3351.461	309	.000	.060	.903	.0489
BSI-18	1490.482	132	.000	.061	.977	.0427
SCL-14	557.444	74	.000	.049	.969	.0325
SCL-K-9	382.378	27	.000	.069	.948	.034

Notes: χ^2^ = Chi-square; df = Degrees of Freedom; RMSEA = Root-Mean-Square-Error-of-Approximation; CFI = Comparative-Fit-Index; SRMR = Standardized-Root-Mean-Square-Residual; ^a^ = in a sample size of N > 250 the Cut-off is 0.06, in N < 250 the Cut-off is 0.08 [22]. N = 2727.

Moreover, the assessment of the BSI scale structure in patients with affective disorders did not reveal a homogenous picture. The RMSEA (0.06) and the SRMR value (0.05) were outside the acceptable range, whereas the CFI (0.83) was outside the cut-off value of ~ 0.95 (see Table 3).

Furthermore, the six-dimensional scale structure of the SCL-27 was not adequately confirmed in patients with affective disorders, although the RMSEA (0.06) and the CFI (0.90) were only marginally outside the range defined by the cut-off values. The SRMR (0.05) was within the accepted range (see Table 3). With regard to the BSI-18, satisfactory cut-off values were confirmed. The postulated scale structure of the SCL-14 was replicated using confirmatory factor analysis, as all three indices were within the expected range (see Table 3).

Additionally, the one-dimensional scale structure of the SCL-K-9 was supported with confirmatory factor analysis; although the RMSEA (0.07) was marginally above the cut-off values, the other two indices lay within the expected range (see Table 3). We provided additional material on the covariance matrix for the SCL-90-R and their short versions of the confirmatory factor analyses (please see Additional files 1, 2, 3, 4, 5 and 6 as PDF).

### Equivalence of the short forms with the SCL-90-R

With regard to the equivalence of the short versions and the SCL-90-R in patients with affective disorders, paired t-tests showed significant mean value differences in all symptom scales and the global GSI index between the SCL-90-R scales and the corresponding scales of the BSI, SCL-27, BSI-18, SCL-14, and SCL-K-9 (see Table 4). To assess the practical significance, effect sizes of the calculated mean value differences were also calculated. Excluding “psychotic” and “insecurity in social contacts” (comparing SCL-90-R and BSI), “compulsiveness” and “depression” (comparing SCL-90-R and SCL-27), “depression” and “phobic anxiety” (comparing SCL-90-R and –SCL-14), and the global GSI index (comparing SCL-90-R and SCL-K-9), the scale differences showed small effect sizes (ES d < 0.20) (see Table 4). A medium effect was only found for the comparison of the total GSI value of the SCL-90-R and the SCL-K-9 (d = 0.62). Consistently high correlations were found between the subscales of the SCL-90-R and the subscales of the investigated short forms (r = 0.85 - 0.98).

**Table 4:** Means (M) and standard deviations (SD) of the 9 scales and the GSI of the SCL-90-R and the short versions: BSI, SCL-27, BSI-18, SCL-14, SCL-K-9 and the results of the t-tests, effect sizes and correlations in patients with affective disorders

	**SCL-90-R**			**BSI**				
**Scale**	**M**	**SD**	**Scale**	**M**	**SD**	**t**	**ES**	**r**
SOMA	1.12	0.74	SOMA	1.00	0.75	25.37*	0.16	0.95
OCD	1.57	0.81	OCD	1.74	0.92	-34.65*	-0.20	0.96
INT	1.40	0.87	INT	1.61	0.99	-33.18*	-0.23	0.95
DEPR	1.73	0.83	DEPR	1.69	0.97	6.07*	0.05	0.94
ANX	1.27	0.80	ANX	1.41	0.85	-30.10*	-0.17	0.96
HOST	0.89	0.71	HOST	0.95	0.71	-19.73*	-0.08	0.98
PHOB	0.89	0.85	PHOB	0.99	0.91	-24.69*	-0.11	0.98
PARA	1.13	0.82	PARA	1.20	0.88	-22.80*	-0.08	0.98
PSYC	0.83	0.63	PSYC	1.07	0.82	-37.22*	-0.32	0.93
GSI	1.26	0.64	GSI	1.30	0.67	-22.63*	-0.07	0.99
	**SCL-90-R**			**SCL-27**				
SOMA	1.12	0.74	VEG	1.03	0.78	12.51*	0.12	0.88
OCD	1.57	0.81	DYS	1.83	1.04	-26.49*	-0.28	0.88
INT	1.40	0.87	SOP	1.43	1.04	-3.92*	-0.03	0.94
DEPR	1.73	0.83	DEP	1.40	0.93	34.28*	0.37	0.85
PHOB	0.89	0.85	AGO	0.86	0.86	5.07*	0.04	0.94
PARA	1.13	0.82	MIS	1.13	0.90	1.14^a^	0.01	0.95
GSI	1.26	0.64	GSI	1.27	0.71	-4.04*	-0.02	0.97
				**BSI 18**				
SOMA			SOM	1.01	0.80	20.71*	0.14	0.94
DEPR			DEP	1.69	0.97	6.07*	0.04	0.94
ANX			ANX	1.44	0.87	-37.90*	-0.20	0.97
GSI			GSI	1.38	0.74	-24.44*	-0.17	0.94
	**SCL-90-R**			**SCL-14**				
SOMA	1.12	0.74	SOM	1.18	0.97	-6.27*	-0.07	0.88
DEPR	1.73	0.83	DEP	1.93	1.00	-28.04*	-0.22	0.94
PHOB	0.89	0.85	PHO	0.65	0.87	37.15*	0.28	0.93
GSI	1.26	0.64	GSI	1.35	0.75	-15.36*	-0.13	0.92
	**SCL-90-R**			**SCL-K-9**				
GSI	1.26	0.64	GSI	1.71	0.83	-64.25*	-0.62	0.91

Notes: Abbreviations of the SCL-90-R, BSI and BSI-18: SOMA = Somatization; OCD = obsessive-compulsive; INT = interpersonal sensitivity; DEPR = Depression; ANX = Anxiety; HOST = Hostility; PHOB = Phobic Anxiety; PARA = Paranoid Ideation; PSYC = Psychoticism; GSI = Global Severity Index; Abbreviations of the SCL-27: DEP = Depressive symptoms; DYS = Dysthymic symptoms; VEG = Vegetative symptoms; AGO = Agoraphobic symptoms; SOP = Symptoms of social phobia; MIS = Symptoms of mistrust; GSI = Global Severity Index; Abbreviations of the SCL-14: DEP = Depression; PHO = Phobic Anxiety; SOM = Somatization; GSI = Global Severity Index; Abbreviations of the SCL-K-9: GSI = Global Severity Index. * Correlations are significant at p < 0.05 (2-tailed). ^a^ Correlation is not significant. 2637 ≤ N ≤ 2638.

### Convergent and discriminant validity of the short forms compared to the SCL-90-R

The assessment of convergent validity showed a statistically significant correlation between the BDI total score and the SCL-90-R depression scale in patients with affective disorders (r = 0.80, see Table 5). The BDI total score was slightly less correlated with the BSI depression scale (r = 0.77); however, it was still fairly high overall. The depression scale of the SCL-27 showed a significantly high correlation with the BDI total score (r = 0.71). Similar to the BSI, the BSI-18 also showed a high correlation with the BDI total score (r = 0.77). The correlation between the BDI total score and the SCL-14 depression scale was also very high. The depression item of the SCL-K-9 was correlated with the BDI total score and, as expected, displayed a medium correlation of r = 0.51 (see Table 5).

**Table 5:** Convergent validity: (Product–moment-Correlations) between the scales of the SCL-90-R, BSI, SCL-27, BSI-18, SCL-14 and SCL-K-9 and the total score of the Beck-Depression-Inventory (BDI, Hautzinger et al., 1995) in patients with affective disorders

**SCL-90-R**		**BSI**		**SCL-27**		**BSI-18**		**SCL-14**		**SCL-K-9**	
**Scale**	**r**	**Scale**	**r**	**Scale**	**r**	**Scale**	**r**	**Scale**	**r**	**Scale**	**r**
SOMA	0.51**	SOMA	0.50**	VEG	0.50**	SOMA	0.49**	SOMA	0.41**		
OCD	0.68**	OCD	0.66**	DYS	0.61**						
INT	0.65**	INT	0,64**	SOP	0.60**						
**DEPR**	0.80**	**DEPR**	0.77**	**DEP**	0.71**	**DEPR**	0.77**	**DEPR**	0.79**	**itemD**	0.51**
ANX	0.61**	ANX	0.58**			ANX	0.59**				
HOST	0.49**	HOST	0.49**								
PHOB	0.51**	PHOB	0.52**	AGO	0.52**			PHOB	0.41**		
PARA	0.52**	PARA	0.52**	MIS	0.50**						
PSYC	0.65**	PSYC	0.66**								
GSI	0.77**	GSI	0.77**	GSI	0.75**	GSI	0.74**	GSI	0.74**	GSI	0.69**

Notes: Abbreviations of the SCL-90-R, BSI and BSI-18: SOMA = Somatization; OCD = obsessive-compulsive; INT = interpersonal sensitivity; DEPR = Depression; ANX = Anxiety; HOST = Hostility; PHOB = Phobic Anxiety; PARA = Paranoid Ideation; PSYC = Psychoticism; GSI = Global Severity Index; Abbreviations of the SCL-27: DEP = Depressive symptoms; DYS = Dysthymic symptoms; VEG = Vegetative symptoms; AGO = Agoraphobic symptoms; SOP = Symptoms of social phobia; MIS = Symptoms of mistrust; GSI = Global Severity Index; Abbreviations of the SCL-14: DEP = Depression; PHO = Phobic Anxiety; SOM = Somatization; GSI = Global Severity Index; Abbreviations of the SCL-K-9: GSI = Global Severity Index. ** Correlations are significant at p < 0.01 (2-tailed). 2601 ≤ N ≤ 2689.

With regard to discriminant validity, theoretically expected low correlations were not found, but only for the SCL-14 and the BSI-18 we found lower correlations (see Table 5). For example, there was a medium correlation between the somatisation scale of the SCL-90-R, BSI, SCL-27, and BSI-18 and the BDI total score (r = 0.49 to r = 0.51). The construct-unrelated scales of the SCL-14, however, showed low correlations with the BDI total score (r = 0.41 “SCL-14 phobic anxiety”, r = 0.41 “SCL-14 somatisation”).

An assessment of the correlation coefficient using Fisher`s z-test revealed that due to the large sample size, even small deviations of 0.01 resulted in statistically significant results.

### Sensitivity to change of the short versions compared to the SCL-90-R

An assessment of the sensitivity to change showed only one larger deviation for the SCL-27 of pre-post effect sizes (see Table 6) compared to the effect size of the SCL-90-R. The others ranged between d = 0.67 and d = 0.68 in patients with affective disorders. The second substantial difference was found in the value of the effect sizes for the “phobic anxiety” subscale of the SCL-90-R (d = 0.39), BSI (d = 0.41), and SCL-14 (d = 0.28). For all other scales, the comparisons showed only minor differences in calculated effect sizes (see Table 6).

**Table 6:** Mean pre-post effect sizes (d) for scales of the SCL-90-R and the short versions BSI, SCL-27, BSI-18, SCL-14 and SCL-K-9 in patients with affective disorders

**SCL-90-R**		**BSI**		**SCL-27**		**BSI-18**		**SCL-14**		**SCL-K-9**	
**Scale**	**d**	**Scale**	**d**	**Scale**	**d**	**Scale**	**d**	**Scale**	**d**	**Scale**	**d**
SOMA	0.37	SOMA	0.34	VEG	0.35	SOMA	0.33	SOMA	0.29		
OCD	0.61	OCD	0.58	DYS	0.54						
INT	0.52	INT	0.54	SOP	0.52						
**DEPR**	**0.72**	**DEPR**	**0.67**	**DEP**	**0.52**	**DEPR**	**0.67**	**DEPR**	**0.68**		
ANX	0.47	ANX	0.51			ANX	0.51				
HOST	0.37	HOST	0.40								
PHOB	0.39	PHOB	0.41	AGO	0.37			PHOB	0.28		
PARA	0.46	PARA	0.47	MIS	0.40						
PSYC	0.44	PSYC	0.43								
GSI	0.62	GSI	0.63	GSI	0.59	GSI	0.61	GSI	0.59	GSI	0.63

Notes: Abbreviations of the SCL-90-R, BSI and BSI-18: SOMA = Somatization; OCD = obsessive-compulsive; INT = interpersonal sensitivity; DEPR = Depression; ANX = Anxiety; HOST = Hostility; PHOB = Phobic Anxiety; PARA = Paranoid Ideation; PSYC = Psychoticism; GSI = Global Severity Index; Abbreviations of the SCL-27: DEP = Depressive symptoms; DYS = Dysthymic symptoms; VEG = Vegetative symptoms; AGO = Agoraphobic symptoms; SOP = Symptoms of social phobia; MIS = Symptoms of mistrust; GSI = Global Severity Index; Abbreviations of the SCL-14: DEP = Depression; PHO = Phobic Anxiety; SOM = Somatization; GSI = Global Severity Index; Abbreviations of the SCL-K-9: GSI = Global Severity Index. d = effect size. 2290 ≤ N ≤ 2294.

## Discussion

This study investigated the psychometric properties of the German version of the SCL-90-R [18] and its short versions, the BSI [25], SCL-27 [7], BSI-18 [6], SCL-14 [8], and SCL-K-9 [10], in a large sample of 2,727 inpatients with affective disorders.

With regard to reliability, no significant difference regarding the internal consistencies of the SCL-90-R and its short versions was expected. The calculated internal consistencies of the short forms were within a satisfactory range compared to the SCL-90-R and corresponded to reliability values reported by Derogatis [6], except for the BSI aggression scale (α=0.67). As expected, the reliability values were decreased with decreasing item numbers but highest for the GSI of the SCL-90-R and its short versions.

Regarding the factorial validity, the originally postulated scale structure of the SCL-90-R by Derogatis [4] was not supported in this study on patients with affective disorders, which is in line with numerous previous studies [2,26,27]. Furthermore, the assessment of the BSI scale structure showed an insufficient model fit, leading to a rejection of the postulated model. Fit indices of the SCL-27 scale structure supported a better fit of the scale structure but were not regarded as satisfactory. These results were largely in agreement with findings of Hardt et al. [7]. The BSI-18 also showed largely satisfactory results regarding its factorial validity in patients with affective disorders.

In line with the findings of Harfst et al. [8], confirmatory factor analysis of the SCL-14 supported the postulated scale structure in the present study. The one-dimensional scale structure of the SCL-K-9 and the dimensional scale structure of the BSI-18 were also supported in confirmatory factor analysis. Overall, a good model fit was only reported for the BSI-18, SCL-14 and SCL-K-9 short forms. Therefore, the factorial validity of the SCL-90-R, BSI and SCL-27 remains questionable.

An assessment of the equivalence of scale values of the short versions and the SCL-90-R in patients with affective disorders showed, as expected, no significant differences. The only exception was the divergence between the GSI of the SCL-90-R and the SCL-K-9, which showed a medium effect size (d = 0.62). Overall, it can be concluded that the symptom severity values of the SCL-90-R scales are comparable to values of the short versions.

The convergent validity of the SCL-90-R and its short versions can be regarded as satisfactory. There were consistently significant correlations with at least large effect sizes. These results support the suitability of the SCL-90-R short versions as a screening instrument to cover a wide range of psychopathological symptoms in patients with affective disorders without a substantial loss of information.

Similar to the findings of the factorial validity of the SCL-90-R and the majority of its short versions, the discriminant validity was also not regarded as satisfactory. The only exception was the SCL-14 [8], in which the two subscales “somatisation“ and “phobic anxiety“correlated with the BDI total score at r = 0.41. Although this correlation was still of at least medium effect size, it was nonetheless substantially lower in comparison to the scales of the short versions and the SCL-90-R. Therefore, the discriminant validity of the SCL-90-R and its short versions in patients with affective disorders cannot be regarded as satisfactory.

The assessment of sensitivity to change was expected to show no marked difference between the SCL-90-R and its short versions in patients with affective disorders. The results of sensitivity to change showed only small differences in the calculated effect size comparisons of the different scales and the GSI of the SCL-90-R and its short versions BSI, SCL-27, BSI-18, SCL-14 and SCL-K-9 and were thus regarded as non-significant. Thus, the short versions BSI, SCL-27, BSI-18, SCL-14, and SCL-K-9 are suitable for patients with affective disorders and are similarly appropriate to detect sensitivity to change as the SCL-90-R. Moreover, the results showed, as expected, that effect sizes of the SCL-90-R depression scale and its short versions were highest compared to other scales (e.g., the somatisation scale), excluding the SCL-27 [7]. This finding supports the applicability of the SCL-90-R and its short versions as an outcome measure instruments in patients with affective disorders.

Overall, it can be concluded that the short versions are a more economic variant of the SCL-90-R, although for the SCL-90-R, only the interpretation of the total score can be recommended. This finding is in line with empirical findings from other studies [28,29], which also recommend the use of the SCL-90-R as a screening instrument using the global index. However, the present study shows that the more economic short versions can also be applied with similar psychometric properties, particularly validity, in patients with affective disorders.

Furthermore, the reported empirical findings show that the three-dimensional factor structure of the BSI-18 and the SCL-14 [8] was replicated. Therefore, from a psychometric perspective, it can be concluded that the BSI-18 and the SCL-14 are not only a more economic alternative but can also be regarded as a psychometric improvement. The SCL-K-9, with only 9 items, does not have a broad range of measurement and only limitedly contributes to the assessment of a wide range of psychopathological symptoms within the context of a heterogeneous clinical sample. Moreover, the assessment of changes within the therapy process, an important criterion for outcome instruments, is also fulfilled across all short versions.

A number of limitations of this study need to be noted. The psychometric assessment was based on a data set in which values of the short versions BSI, SCL-27, BSI-18, SCL-14, and SCL-K-9 were calculated post-hoc and may be intercorrelated. Our results showed high correlations between the scores of the SCL-90-R and its short versions. If we had advised the patients to fill out the versions separately, this high intercorrelation may have decreased. Nonetheless, this procedure appeared feasible against the background of missing comparable studies on patients with affective disorders. Further psychometric assessments using individual applications of the short versions BSI, SCL-27, BSI-18, SCL-14, and SCL-K-9 should be carried out to support the reported findings of this study. Regarding the generalisability of our results, we compared the SCL-90-R and its short versions in a widely representative sample of inpatients with affective disorders undergoing psychotherapeutic treatment in Germany. There is a need for the further psychometric evaluation of patients with affective disorders in other settings (e.g., outpatients, day clinics).

## Conclusions

In summary, based on all empirical findings, the **BSI-18** can be recommended as an economic variant and clinically meaningful instrument to measure symptom severity in patients with affective disorders.

## Competing interests

The authors declare that they have no competing interests.

## Authors’ contributions

UP carried out the study, analysed the data and drafted the first version of the manuscript. DON, HS, FP, and CB participated in the design of the study and helped to draft the manuscript. SA conceived of the study and helped to draft the manuscript. All authors read and approved the final manuscript.

## Pre-publication history

The pre-publication history for this paper can be accessed here:

http://www.biomedcentral.com/1471-244X/13/104/prepub

## Supplementary Material

Additional file 1 AMOS Graphics SCL-90-R. Click here for file

Additional file 2 AMOS Graphics BSI. Click here for file

Additional file 3 AMOS Graphics SCL-27. Click here for file

Additional file 4 AMOS Graphics BSI-18. Click here for file

Additional file 5 AMOS Graphics SCL-14. Click here for file

Additional file 6 AMOS Graphics SCL-K9. Click here for file
