# Supplementary figures and images for: Comparative psychometric analyses of the SCL-90-R and its short versions in patients with affective disorders
Source: BMC Psychiatry. 2013 Mar 28;13:104. doi: 10.1186/1471-244X-13-104 (PMC3626675; doi:10.1186/1471-244X-13-104)

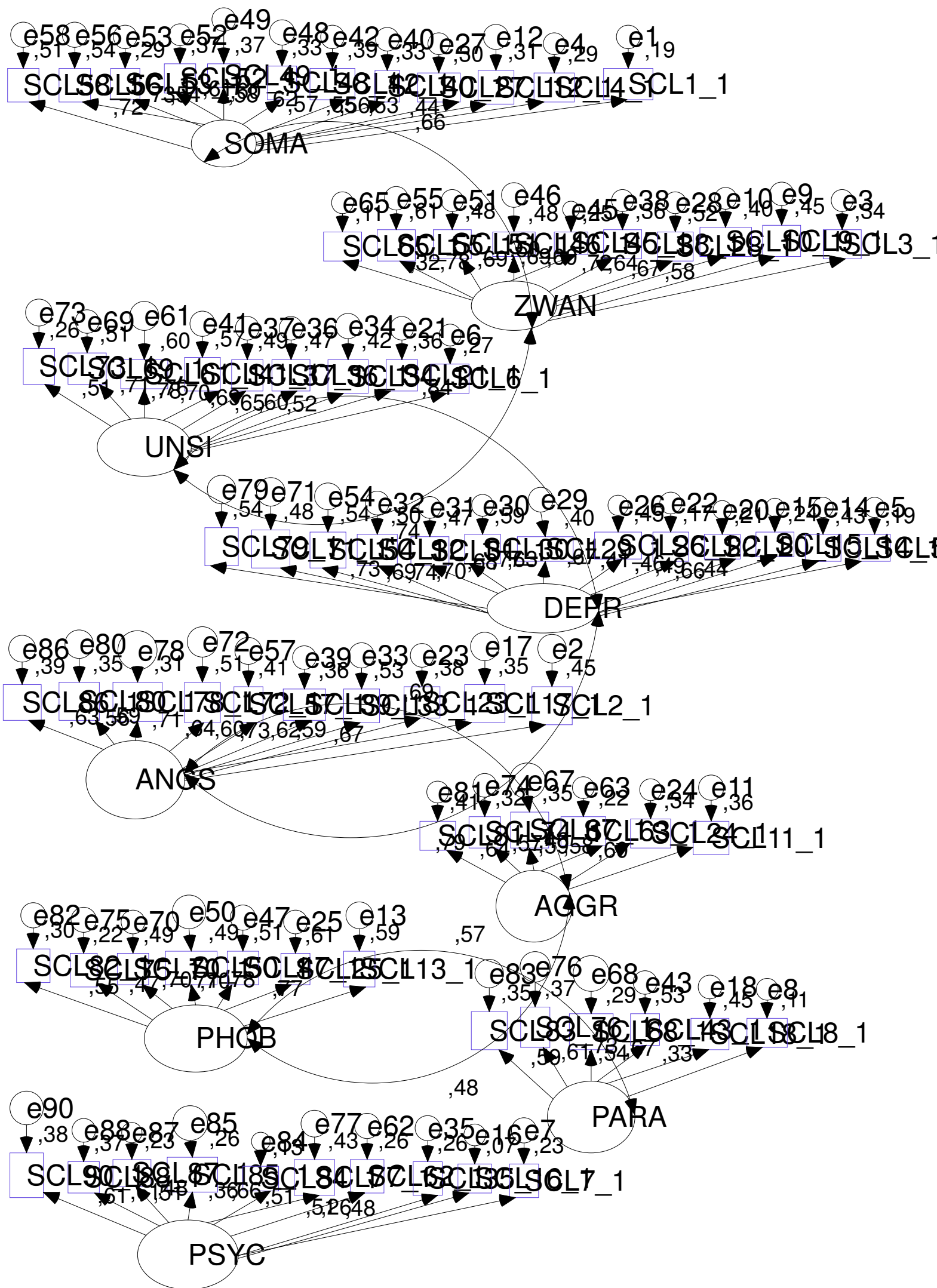

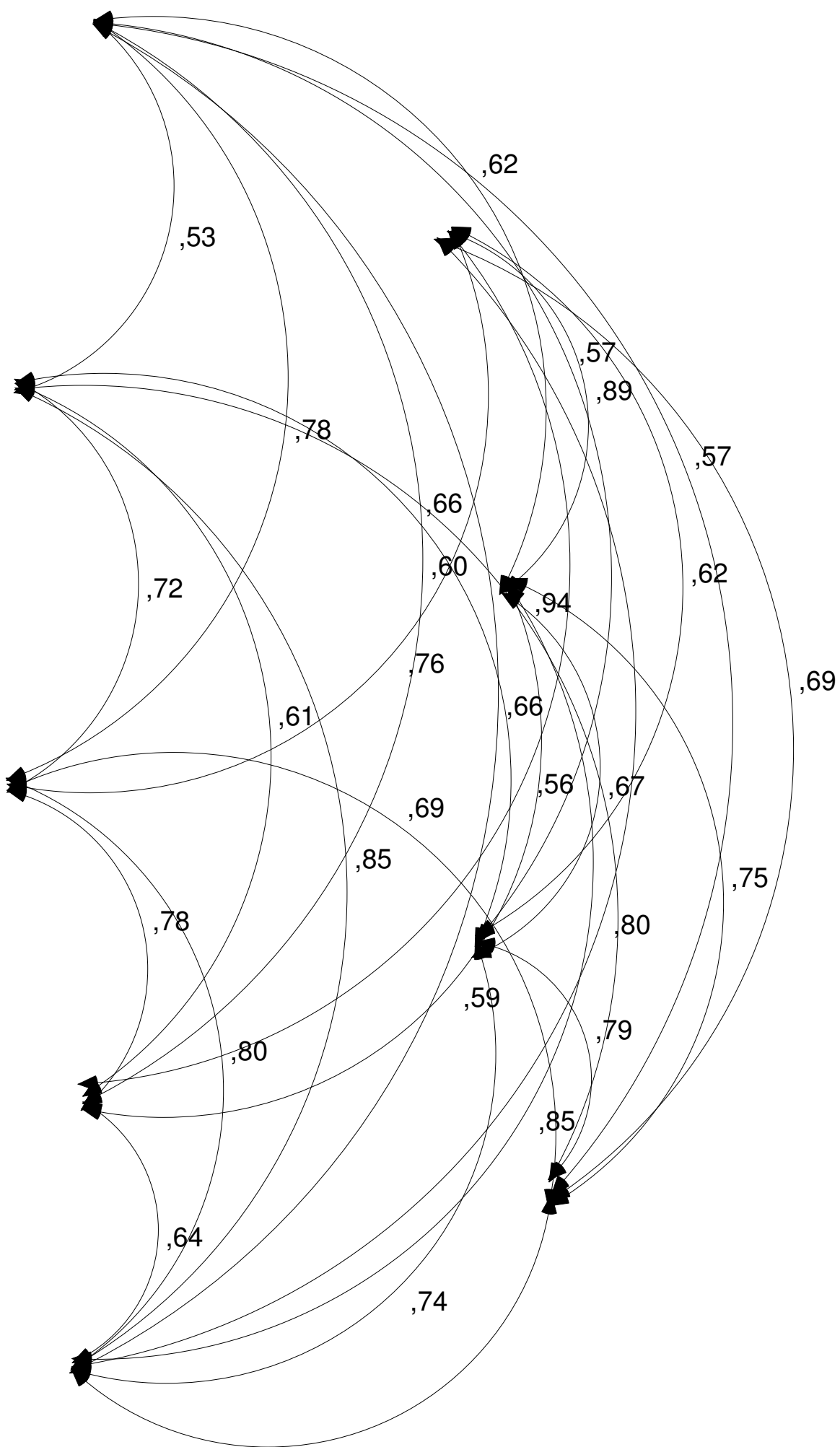

Supplement: Additional file 1 — AMOS Graphics SCL-90-R. [file 1471-244X-13-104-S1.pdf]

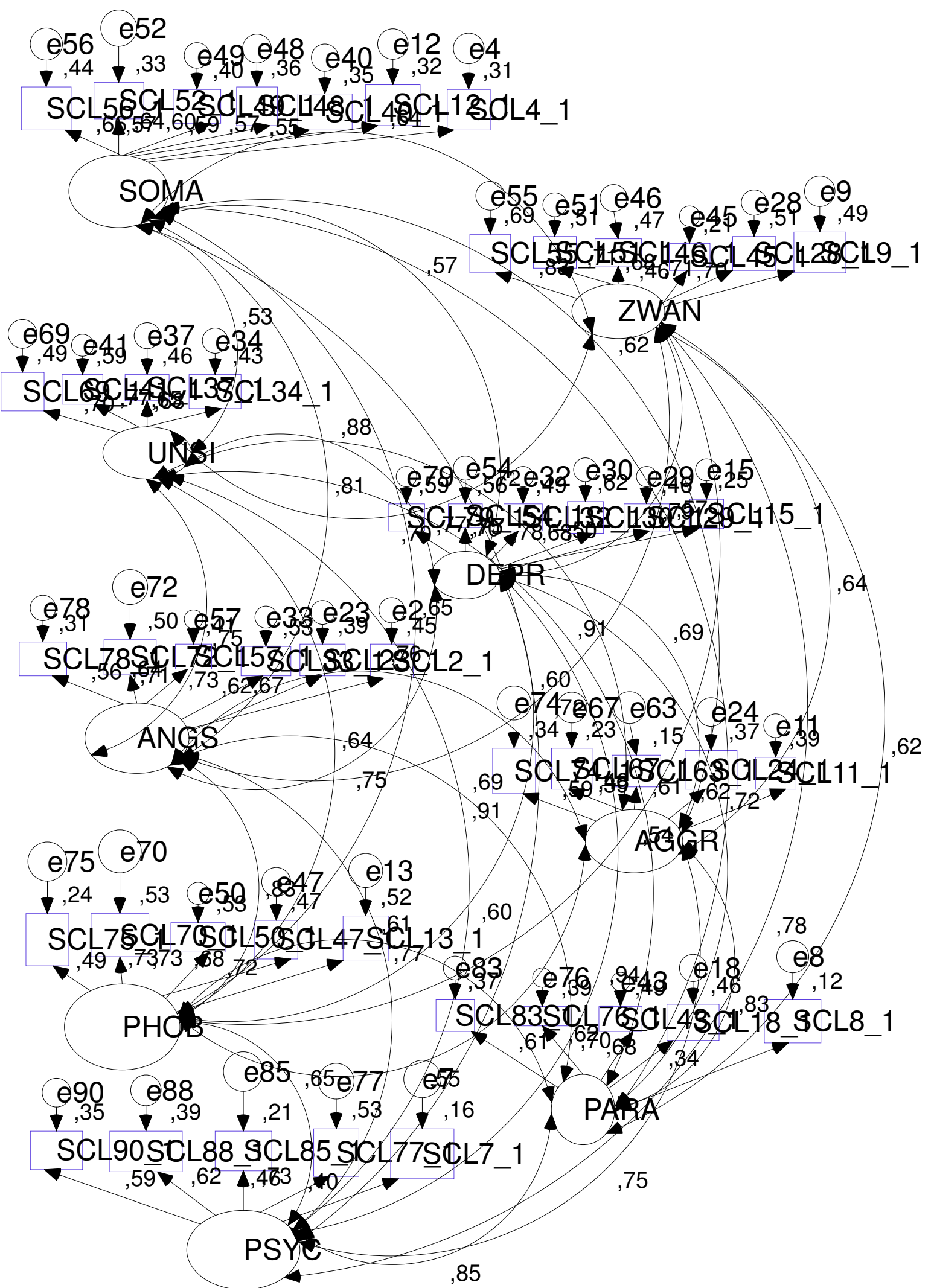

Supplement: Additional file 2 — AMOS Graphics BSI. [file 1471-244X-13-104-S2.pdf]

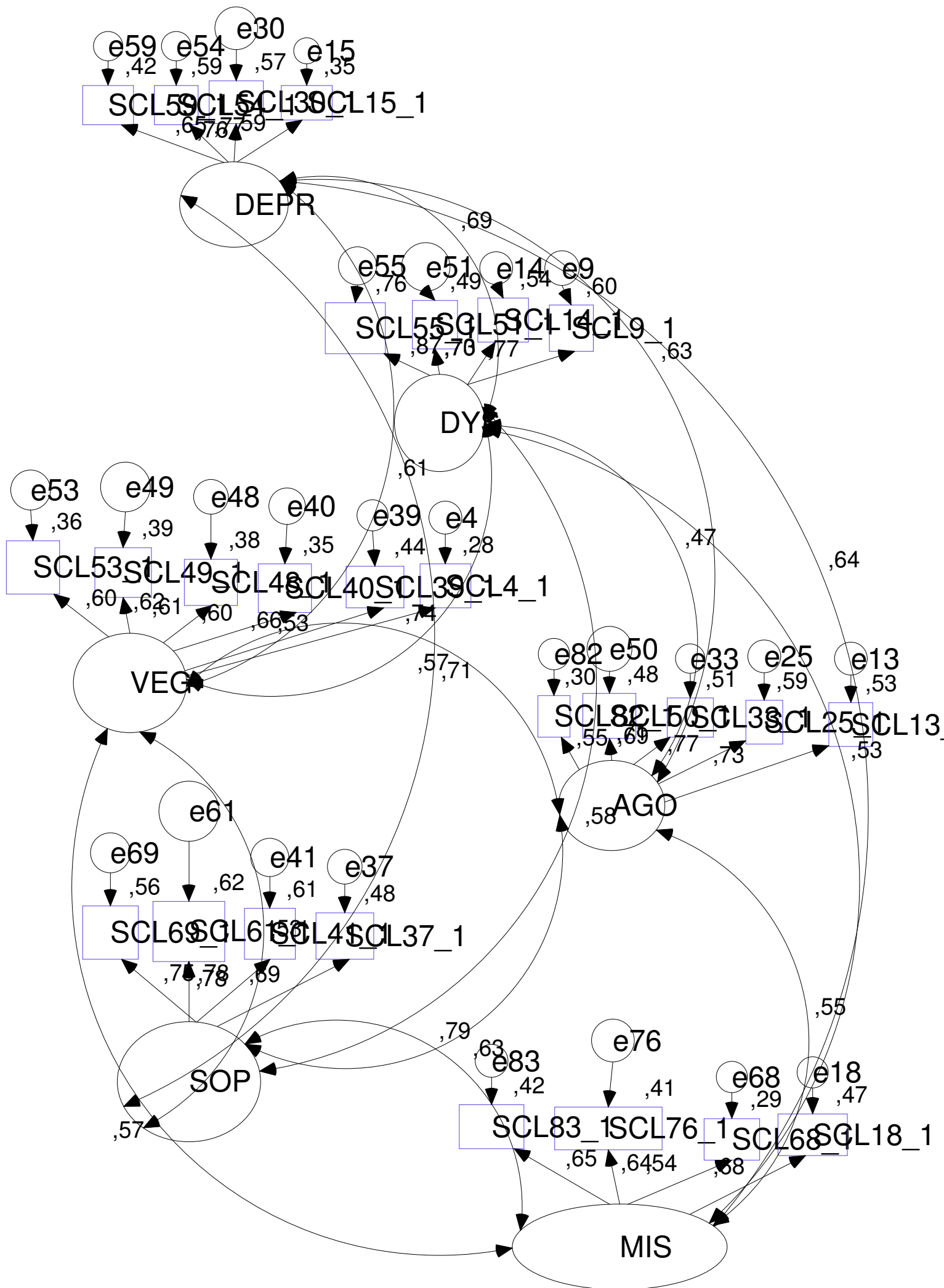

Supplement: Additional file 3 — AMOS Graphics SCL-27. [file 1471-244X-13-104-S3.pdf]

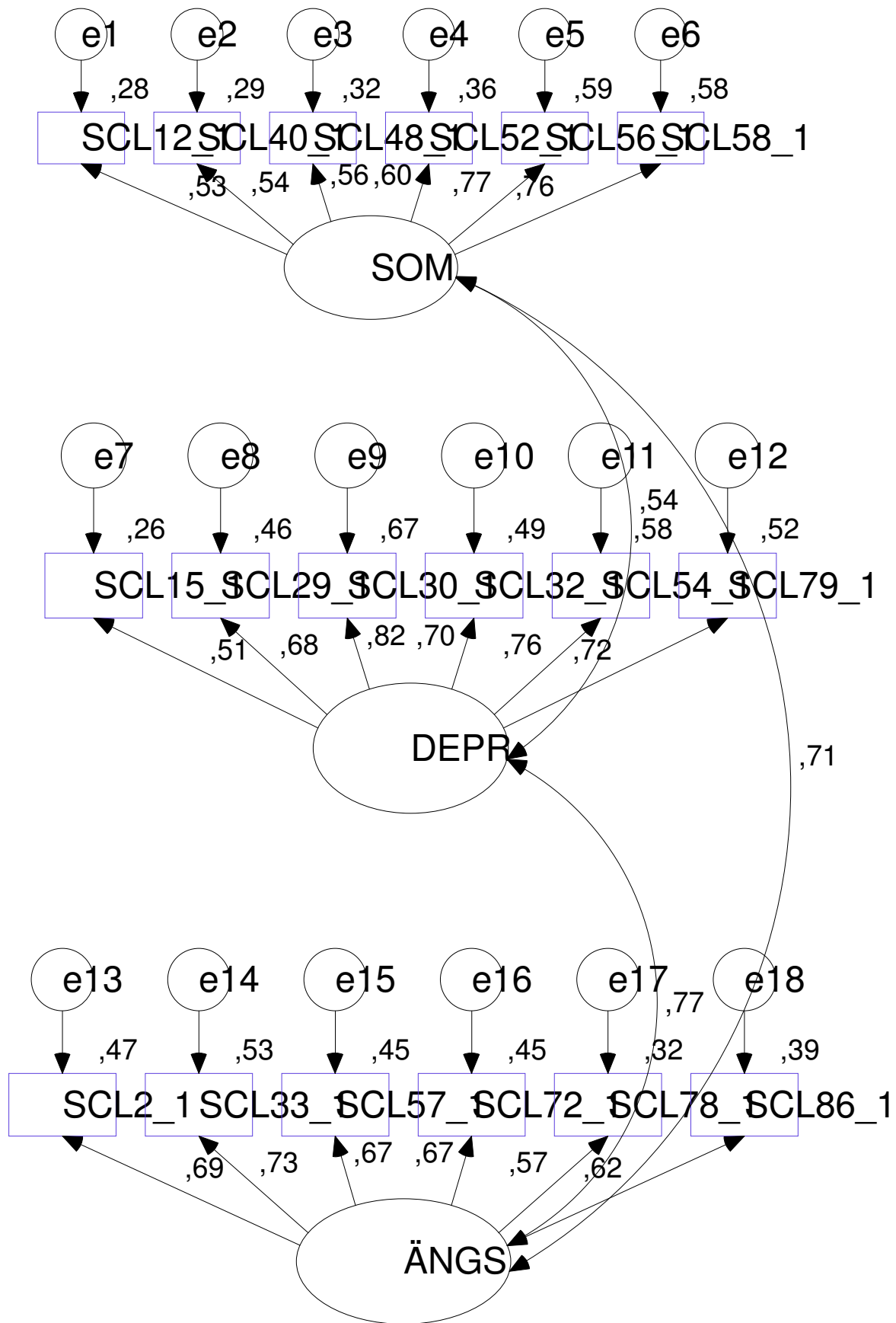

Supplement: Additional file 4 — AMOS Graphics BSI-18. [file 1471-244X-13-104-S4.pdf]

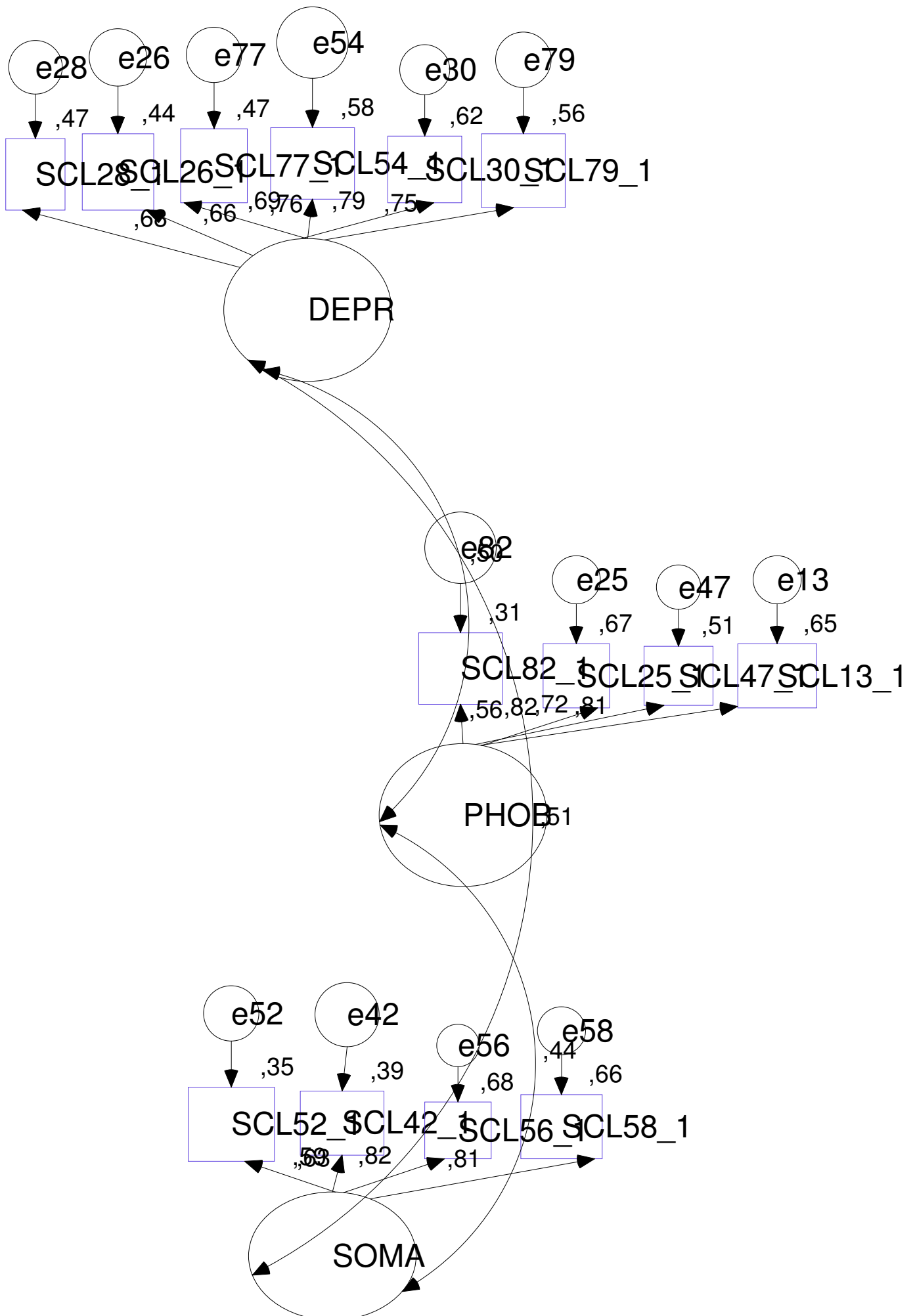

Supplement: Additional file 5 — AMOS Graphics SCL-14. [file 1471-244X-13-104-S5.pdf]

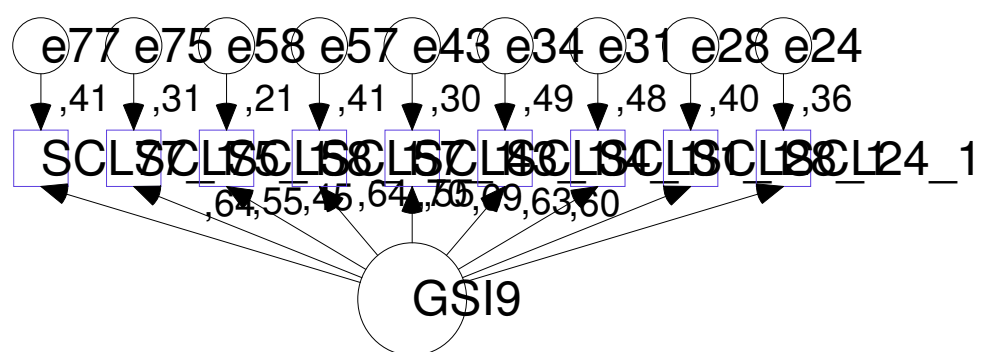

Supplement: Additional file 6 — AMOS Graphics SCL-K9. [file 1471-244X-13-104-S6.pdf]
